# Capability of Copper Hydroxy Nitrate (Cu_2_(OH)_3_NO_3_) as an Additive to Develop Antibacterial Polymer Contact Surfaces: Potential for Food Packaging Applications

**DOI:** 10.3390/polym15071661

**Published:** 2023-03-27

**Authors:** Xiomara Santos, Juana Rodríguez, Francisco Guillén, Javier Pozuelo, J. M. Molina-Guijarro, Diogo Videira-Quintela, Olga Martín

**Affiliations:** 1 Department of Materials Science and Engineering and Chemical Engineering, Higher Polytechnic School, Carlos III University of Madrid, Avenida Universidad 30, 28911 Leganés, Spain; xsantos@ing.uc3m.es (X.S.); jpozue@uc3m.es (J.P.); 2 Department of Biomedicine and Biotechnology, Faculty of Pharmacy, University of Alcalá, Ctra. Madrid-Barcelona km 33.6, 28871 Alcalá de Henares, Spain; juana.rodriguez@uah.es (J.R.); francisco.guillen@uah.es (F.G.); josemanuel.molina@uah.es (J.M.M.-G.); 3 Department of Analytical Chemistry, Physical Chemistry and Chemical Engineering, Faculty of Pharmacy, University of Alcalá, Ctra. Madrid-Barcelona km 33.6, 28871 Alcalá de Henares, Spain

**Keywords:** PLA, LDPE, copper(II) hydroxy nitrate, bactericidal activity, food contact material, antibacterial surface, food packaging

## Abstract

The globalization of the market, as well as the increasing world population, which require a higher demand for food products, pose a great challenge to ensure food safety and prevent food loss and waste. In this sense, active materials with antibacterial properties are an important alternative in the prolongation of shelf life and ensuring food safety. In this work, the ability of copper(II) hydroxy nitrate (CuHS) to obtain antibacterial films based on low density polyethylene (LDPE) and polylactic acid (PLA), was evaluated. The thermal properties of the composites, evaluated using thermogravimetric analysis (TGA) and differential scanning calorimetry (DSC), showed that the concentrations of added CuHS do not particularly change these characteristics with respect to the neat polymer matrix films. The mechanical properties, determined using dynamic mechanical analysis (DMTA), indicate a small increase in the brittleness of the material in PLA-based composites. The antibacterial properties against *Listeria monocytogenes* and *Salmonella enterica* were evaluated using a surface contact test, and a bacterial reduction of at least 8 to 9 logarithmic units for the composites with 0.3% CuHS, both in LDPE and PLA and against both bacteria, were achieved. The reusability of the composite films after their first use demonstrated a higher stability against *Listeria monocytogenes*. The migration and cytotoxicity of the composites loaded with 0.3% CuHS was evaluated, demonstrating the safety of these materials, which reinforces their potential use in food packaging applications.

## 1. Introduction

According to the Food and Agriculture Organization of the United Nations (FAO), in 2011 it was estimated that approximately one third of the food produced globally for human consumption was lost or wasted, corresponding to 1.3 billion tons of food each year [1]. This has an enormous impact on food security, biodiversity, and the environment. In fact, food loss and waste are the third largest source of greenhouse gas emissions [2].

Another related problem is the emergence of foodborne diseases, which remain a common and serious threat to public health worldwide and a major cause of morbidity and mortality. According to the World Health Organization (WHO) report published in 2015, an estimated 600 million people (almost 1 in 10 people worldwide) suffer from diseases caused by contaminated food, resulting in 420,000 deaths each year [3].

Among the microorganisms responsible, norovirus, *Salmonella*, *Campylobacter* spp., *Listeria monocytogenes*, *Staphylococcus aureus* and *Escherichia coli* O157:H7 were identified as the main agents [4,5]. Specifically, *Salmonella* and *Listeria* are two of the pathogens with the greatest impact on health and the economy worldwide [5,6,7,8,9]. Apart from the health aspect, these diseases also affect socio-economic development [3]. In a 2019 report by the World Bank, it was estimated that a total of USD110 billion per year is lost in productivity and medical costs due to the treatment of foodborne diseases [10].

Among the various causes responsible for food waste and foodborne diseases, inadequate transport and storage conditions tend to favor bacterial, fungal or insect attacks, leading to food spoilage and a shorter shelf-life. There is also a risk of microbial contamination during processing and unsafe food handling [1].

These problems could be avoided through the application of new packaging technologies. In fact, according to the American Institute for Packaging and the Environment, 20 to 25% of food waste can be avoided through the application of food packaging technologies [11]. Furthermore, as mentioned by the FAO, food loss and waste can be reduced using appropriate packaging [1]. This can be achieved by inhibiting the microbial growth responsible for food spoilage and, at the same time, inhibiting the growth of the pathogenic microorganisms associated with the occurrence of foodborne diseases. To this end, the development of antimicrobial active packaging systems constitutes an important alternative for prolonging the freshness and quality of food, which translates into an increase in shelf life and food safety for consumers [6,12,13].

Films active against different bacteria and contaminating microorganisms constitute a reinforcement of food safety for the consumer. Adding molecules or nanoparticles with antibacterial properties to common food packaging polymeric matrices provides these systems with a substantial added value for commercial applications [14]. These types of active materials may be effective both during food production and storage, as well as in obtaining active surfaces that prevent possible cross-contamination through cutting boards, knives, and during food processing [4].

Polymer/nanoparticle composite films containing silver [15,16], copper [17,18], zinc oxide [19,20], titanium [21,22,23] and iron [24,25] as inorganic fillers have been studied for their potential applications in the food packaging industry due to their antibacterial properties [12]. Particularly, copper-based compounds have been employed as a biocide due to their great abundance and their wide antimicrobial activity against many pathogens [4,14].

Among the inorganic fillers, layered double hydroxides (LDH), hydroxide double salts (HDS) and basic salts (BS) stand out. These fall into the family of layered materials, as they are composed of stacks of positively charged metal hydroxide layers, balanced by the presence of exchangeable anions in the interlayer spaces [26]. The structures of LDH and HDS are considered to be derived from the layered network of brucite, (Mg(OH)_2_) [27]. The difference between LDH and HDS is that hydroxide double salts are limited to cations of the same valence, as in brucite (usually divalent); whereas, for LDH, a layer charge is generated as a result of the partial substitution of divalent for trivalent cations, which necessitates the presence of charge-balancing anions within the interlayer [27].

These materials have been used as anion exchangers, employed in catalytic processes, or as carriers for antibacterial host molecules [13,27,28,29]. Certain types of LDH, HDS or BS can be antimicrobial compounds on their own, this being related to their ion release properties [30]. Copper(II) hydroxy nitrate (Cu_2_(OH)_3_NO_3_, CuHS) is a BS that is composed of copper(II) hydroxide layers interconnected by nitrate ions [13,27]. Recently, this BS, decorated on fumed silica and incorporated into an LDPE matrix, demonstrated bactericidal activity against *Staphylococcus aureus* and *Salmonella enterica* [13].

Given these proprieties, this study will be focused on the application of a BS, more specifically CuHS, as a direct antibacterial filler to develop active contact surfaces. Two strategies were planned to produce the active materials via direct incorporation of the CuHS: (i) using melt-extrusion with an LDPE matrix, which is one of the most used polymers in the food packaging industry due to its high chemical and thermal stability, which favors its thermal processability and heat-sealing capabilities [31]; and (ii) using solution casting with PLA, which is a polyester that, due to its biodegradability under composting conditions, biocompatibility, and good mechanical properties, is gaining importance in the food packaging industry [6].

The impact of each strategy was evaluated by performing the physicochemical characterization of the developed composites, and later their antibacterial properties against *L. monocytogenes* and *S. enterica*. To ensure the safety of the CuHS to be applied in the food contact applications, the migration of Cu^2+^ ions in simulated food media and the cytotoxicity of the films with the most promising antimicrobial properties were studied to evaluate if these properties were within the allowed limits.

## 2. Materials and Methods

### 2.1. Materials

Copper(II) nitrate (Sigma-Aldrich, Madrid, Spain), ethanol 99.5% (Quimipur, Madrid, Spain), acetic acid glacial (Panreac, Barcelona, Spain), nitric acid (Scharlau, Barcelona, Spain), dichloromethane (Labkem, Barcelona, Spain), sodium nitrate (Sigma-Aldrich, Madrid, Spain), MTT (3-[4,5-dimethylthiazol-2-yl]-2,5-diphenyltetrazolium bromide) dye (Sigma Cat. No. M5655, Sigma-Aldrich, Madrid, Spain), and filter membranes (Magna, Nylon 47 mm 0.45 µm membrane disk, Fisher Scientific, Waltham, MA, USA) were used throughout the study. Low density polyethylene (LDPE, Riblene MP30R, d25 °C = 0.925 g/cm^3^, melting point of 112 °C, Versalis, Milan, Italy) and polylactic acid (PLA, IngeoTM Biopolymer 2003D NatureWorks, Blair, NE, USA) were kindly gifted by EMSUR (Spain). *S. enterica* (ATCC 35664) was obtained from the American Type Culture Collection (ATCC, Manassas, VA, USA) and *L. monocytogenes* was obtained from a sample recovered at the Hospital Universitario Príncipe de Asturias (Alcalá de Henares, Spain).

### 2.2. Methods

#### 2.2.1. CuHS Synthesis

The synthesis of CuHS was carried out as described in previous work [28]. Briefly, 100 mL of a 0.01 M copper(II) nitrate solution in ethanol was heated in a microwave oven (Sharp R-742 (IN)W) for a total of 2 min (in steps of 15 s). The resultant solution, which had the CuHS precipitated, was cooled to room temperature, followed by filtration and subsequent washing three times with distilled water and hot ethanol. Finally, the CuHS was dried in a vacuum oven at 60 mmHg and 90 °C overnight [27].

#### 2.2.2. Preparation of LDPE/CuHS Composite Films

The LDPE/CuHS composites were obtained in a Haake Polylab QC mixer. The 47 g LDPE and weighted quantities of the CuHS (0.1, 0.3, and 0.5 wt.% by polymer weight) were mixed at 40 rpm at a temperature of 120 °C for 10 min. Films were further obtained via compression molding on a Fontune Presses TPB374 hot plate press. A pressure of 100 kN and a temperature of 120 °C were needed to obtain the films in the press. To achieve homogeneity in the thickness of the films, an aluminum frame of 222 × 122 × 0.6 mm (length, width, thickness) was used.

#### 2.2.3. Preparation of PLA/CuHS Composite Films

PLA/CuHS composite films were prepared at room temperature by solution casting using dichloromethane as the solvent. For this purpose, dispersions of CuHS particles (0.1, 0.3 and 0.5% by polymer mass) were obtained in dichloromethane and then PLA was added up to 10% by mass with respect to the solvent. Adequate particle dispersion was achieved using an ultrasonic treatment for 10 min (at intervals of 10 s on, 30 s off). To achieve homogeneity in film thickness, a 1000 μm thick Doctor Blade was used on a glass support. The composite films were dried at room temperature for 24 h and then placed in an oven at 40 °C for 5 days to achieve complete solvent removal [6,32].

#### 2.2.4. Characterization

The morphological characterization was carried out using a field emission scanning electron microscope (FESEM; Hitachi SU-70; Chiyoda, Tokyo, Japan). The accelerating voltage was 6 kV for the CuHS and 10 kV for the composites’ analysis. The crystalline phase was studied using XRD with Philips X’Pert-MPD equipment (Amsterdam, North Holland, The Netherlands). The 2θ scanning range was set between 5° and 80° with a step size of 0.040°, and the current and tension were set at 40 mA and 40 kV, respectively. To perform the assay, the samples were dispersed in acetone. Raman spectroscopy was employed in a Thermo Scientific confocal DXR Raman spectrometer (Waltham, MA, USA). The instrument employed a laser emitting at 532 nm; in addition, it featured a confocal slit size of 25 μm. Samples were analyzed in the range of 50–3500 cm^−1^, with an exposure time of 30 s for each scan.

Thermal characterization was carried out using differential scanning calorimetry (DSC) and thermogravimetric analysis (TGA). The DSC thermograms were performed with METTLER TOLEDO (Greifensee, Zürich, Switzerland) DSC equipment. For the CuHS, a heating flow from 25 to 400 °C at 15 °C/min was used. The heat treatment for the composite films was performed from 25 to 180 °C at a heating rate of 10 °C/min. Two heating–cooling cycles were performed to remove the thermal history of the material. Both analyses were in a nitrogen atmosphere. TGA was carried out on a Perkin Elmer model STA 6000 (Waltham, MA, USA). CuHS was subjected to heating between 50 and 400 °C at 15 °C/min. For the composite films a heating cycle from 50 to 600 °C at a rate of 30 °C/min was used. The tests were performed in an alumina boat under a nitrogen flow rate of 20 mL/min.

The mechanical characterization of the composite films was obtained using tensile tests to obtain typical stress vs. strain curves. These tests were performed on a TA Instrument dynamomechanical tester (DMTA), model Q800 (New Castle, DE, USA). Films of 20 × 2 mm (length, width) were tested with striped clamps and a force ramp of 3 N/min up to 18 N to cause the fracture of the specimen. The study was carried out in isothermal mode at 35 °C.

#### 2.2.5. Antibacterial Assay of CuHS

The antibacterial properties of CuHS against *L. monocytogenes* (Gram-positive) and *S. enterica* (Gram-negative) were carried out by determining the minimum bactericidal concentration (MBC), following a protocol based on ISO 20776-1:2006 [6,33] Briefly, water dispersions of CuHS with concentrations of 0.5, 1, 2, 3 and 4 mg/mL were prepared using an ultrasonic process. Bacteria were cultured on plate count agar (PCA) plates for 24 h at 37 °C. Then, the colonies were picked and suspended in 20 mL of Muller–Hinton broth medium, and shaken for 24 h at 37 °C. The next day, the bacterial concentration was adjusted to 10^8^ colony forming units (CFU)/mL (absorbance between 0.08 and 0.11 at λ = 625 nm), followed by a dilution to 10^7^ CFU/mL. The assay was performed in 96-well plates. The test was conducted during 24 h of shaking at 37 °C in the presence of 100 μL of the CuHS dispersion, 100 μL of the double concentrated Muller–Hinton medium, and 5 μL of the bacteria. After 24 h, 10 μL of the resulting dispersions were inoculated onto PCA plates and cultured for 24 h at 37 °C. Finally, the growth of the bacteria on the PCA plates was evaluated. Similarly, the minimum bactericidal inhibition (MIC) and the MBC were evaluated for the sodium nitrate and copper(II) nitrate salts, with concentrations of 1 to 5 mg/mL. The MIC involved the measurement of the absorbance at 600 nm using a BioteK plate reader.

#### 2.2.6. Antibacterial Assay of Composite Films

The antibacterial activity of the LDPE/CuHS and PLA/CuHS composites was tested against *L. monocytogenes* and *S. enterica*. This assay was performed using the method described in JIS Z 2801 with some modifications, as reported in a previous paper [6,33]. Briefly, both bacteria were grown on PCA plates for 24 h at 37 °C and then a bacterial dispersion in sterile water was made and adjusted to 10^8^–10^9^ CFU/mL. Then, 100 μL of this dispersion was placed in a sterile polypropylene Petri dish, to which films (2 × 2 cm) were placed on top and kept for 24 h at 28 °C. Next, 10 μL of the bacterial suspension under the films was cultured (non-diluted sample), and tenfold dilutions were made and cultured on PCA plates for 24 h at 37 °C. The bacterial colonies were then counted, and the CFU/mL was calculated. The results were expressed as a mean logarithmic (log) reduction with the corresponding standard deviation by comparison with the initial bacterial concentration. The log reduction was determined by calculating the subtraction of the base 10 logarithms of CFU/mL for the initial bacterial concentration and the corresponding base 10 logarithms of CFU/mL for the blanks and the composite films.

#### 2.2.7. Migration Assay

The specific migration test for copper was performed with liquid food simulant media—food simulant C for aqueous and alcoholic foods (10% *v*/*v* ethanol) and food simulant B for acidic foods (3% *v*/*v* acetic acid)—according to Regulation (EU) No. 10/2011 (EC, 2011) and as described in previous works [13,34]. The assay consisted of the total immersion of the films with a surface of approximately 0.04 dm^2^ in the food simulants for 10 days at 40 °C. Subsequently, the films were removed and 1 mL of the media was diluted with Milli-Q water in the presence of 0.5 mL nitric acid (5% *v*/*v*) to complete 10 mL. This dilution was used for the Cu quantification using inductively coupled plasma-optical emission spectroscopy (Varian 720 ICP-OES). The regulation used to determine copper migration establishes a specific migration limit of 5 mg/kg for samples of 6 dm^2^. Therefore, to determine the migration of the copper in our samples (4 dm^2^), once the migration value in mg/dm^2^ was obtained, it was multiplied by 6 to correct our test with respect to what is established in the regulations.

#### 2.2.8. Cytotoxicity Assay

Cytotoxicity of the composite films was evaluated at 24 and 48 h using the Cell Proliferation Kit I (MTT): Colorimetric assay for the non-radioactive quantification of cell proliferation and viability, using HeLa cells seeded in multiwell plates. To determine cell viability, MTT dye was used, which was dissolved to obtain a 5 mg/mL solution in phosphate-buffered saline (PBS). This solution was filtered through a 0.2 μm filter and stored at 2–8 °C. Routinely, MTT stock solution (5 mg/mL) was added to each culture to be tested to equal one-tenth of the original culture volume and incubated for 3 to 4 h. At the end of the incubation period, the medium was removed to work with adherent cells. The converted dye (from MTT to purple formazan via the interaction with the live cells’ dehydrogenase enzymes) was solubilized in dimethyl sulfoxide (DMSO). The absorbance of the converted dye was measured at a wavelength of 570 nm.

#### 2.2.9. Statistical Analysis

All assays were performed using three to five independent replicas, and the results were provided with a mean ± standard deviation. An analysis of variance was carried out using the Tukey HSD test, to determine significant differences at a 5% significance level (*p* < 0.05) using the statistical software JMP Pro 14 (SAS Institute, Cary, NC, USA).

## 3. Results and Discussion

### 3.1. Characterization of the CuHS Filler

**Figure 1:**
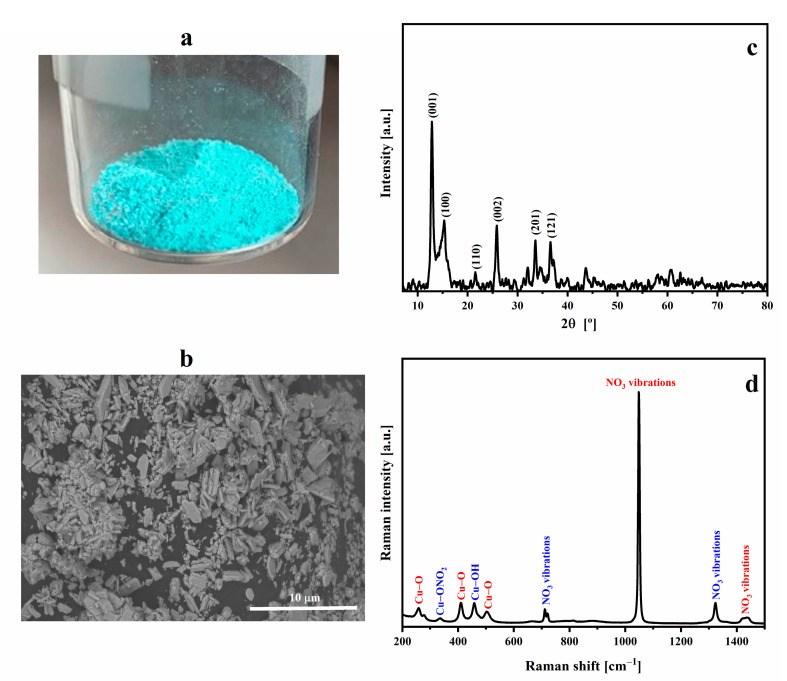
(**a**) Macroscopic aspect; (**b**) FESEM (scale 10 µm); (**c**) XRD with the assignment of the observed planes; and (**d**) Raman spectroscopy of the synthesized CuHS.

Figure 1a shows the macroscopic appearance of a cyan-colored CuHS obtained with the applied microwave-assisted synthesis. The FESEM images of the synthesized CuHS, depicted in Figure 1b, show the heterogeneous shape and size of the CuHS crystals, having average aggregate sizes of less than 5 µm. The EDX analysis evidenced the presence of the elements N, O and Cu at percentages of 8.85, 40.21 and 50.94 wt.%, respectively; no other element in the CuHS structure was observed (Table S1). The obtained wt.% is quite similar to the stoichiometry of the compound with respect to the O and Cu elements (40 and 53%, respectively). Furthermore, the atomic O/Cu ratio (63.68/20.31, as reported in Table S1) is 3.14, a value similar to the theoretical one of 3 [35].

The structure of Cu_2_(OH)_3_NO_3_ has been described using XRD as brucite-like M(OH)_2_ layers in which a quarter of the hydroxyl anions are substituted by nitrate, within which, in turn, the nitrate anion is coordinated, through one of its oxygen atoms, with the Cu^2+^ cation. In general, the structure consists of layers linked by hydrogen bonds between the nitrate and hydroxyl groups [35]. Two polymorphic forms of copper(II) hydroxy nitrate have been studied. The most stable of these is that of the mineral gerhardtite with an orthorhombic form and a metastable structure, which is the one obtained synthetically; a monoclinic type has also been described [35].

In Figure 1c, we present the diffractogram corresponding to the synthesized CuHS. The X‘Pert HighScore program (version 2.2e 2.2.5) was used to index the lines and determine the crystallographic parameters, resulting in a correspondence to a monoclinic P2_1_ [35,36]. According to the reference from the program used to index the peaks, it is a crystalline cell whose a, b and c values are 5.60, 6.08 and 6.93 Å; and α, β and γ of 90.00°, 94.48°, and 90.00°, respectively.

The stability of CuHS at different temperatures was tested in order to confirm its integrity when subjected to the extrusion/pressing processes, performed in this work with an LDPE matrix. Figure S1 shows the diffractogram of Cu_2_(OH)_3_NO_3_, previously subjected to heating for one hour at 120, 140 and 160 °C; the peak corresponding to the (001) plane of CuHS can be observed, demonstrating that at the tested temperatures, the structure of the salt is stable, as reported in works by other authors [36].

The Raman spectrum of CuHS (Figure 1d) gave a similar correlation with the reported vibrational assignment of each of the signals [33]. The bands corresponding to the Cu-O and Cu-OH bonds, and the vibrations of the nitrate group are observed (Figure S2). In addition, a very weak signal is observed near 336 cm^−1^ that could be due to the Cu-ONO_2_ stretching vibration usually present in the structure of natural *gerhardtite* [37].

The thermal characterization of the CuHS is shown in Figure S2. The TGA analysis (Figure S2a) shows a loss of 33% in mass in a single step at 225 °C (T onset). No mass loss is observed due to the presence of water in the structure (around 100 °C), which agrees with the drying procedure performed. The thermal decomposition of CuHS is due to the destruction of its structure through dihydroxylation and the transformation of nitrate ions into nitrogen oxides [27,35]. The expected mass loss for the decomposition of the basic salt to form CuO is 33.8% [27], which is similar to the value obtained experimentally. Only one endothermic process is observed in the DSC (Figure S2b) at 227 °C (T onset). This is related to the decomposition of the salt, which has an associated energy of 784 J/g.

**Figure 2:**
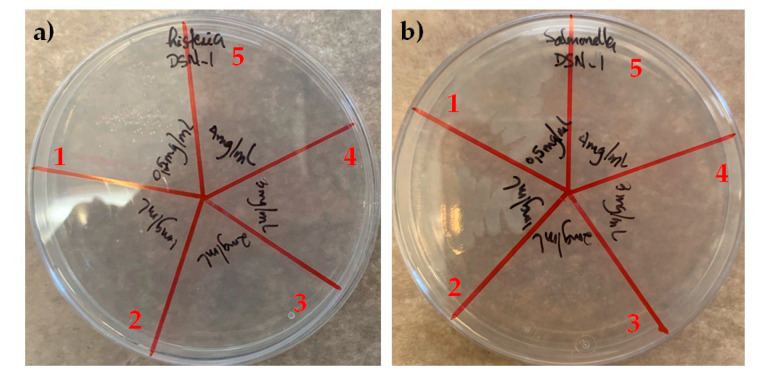
MBC of CuHS against (**a**) *L. monocytogenes*; (**b**) *S. enterica*. The numbers correspond to the following concentrations: 1 corresponds to 0.5 mg/mL; 2 to 1 mg/mL; 3 to 2 mg/mL; 4 to 3 mg/mL; and 5 to 4 mg/mL.

Prior to the incorporation of CuHS as a filler for the composite films’ manufacturing, a first screening of the bactericidal properties of CuHS was tested against *L. monocytogenes* and *S. enterica*. Figure 2 shows that CuHS has good antibacterial capability against the two microorganisms tested. The bactericidal (or MBC) effect of the 1 mg/mL dispersion was sufficient to kill *L. monocytogenes*, while for *S. enterica,* a 2 mg/mL concentration dispersion was required.

Shabestari et al. warns that care should be taken with CuHS because it can decompose at 150–400 °C [28]. According to the results reported in the TGA, the synthesized CuHS does not start its degradation until 225 °C. To evaluate whether exposure to temperature limits the biocidal properties of CuHS, the MBC of CuHS subjected to different temperatures (400 and 600 °C for 1 h) was determined. Figure S3 shows the results obtained against *L. monocytogenes,* and in all cases, the MBC is 1 or 2 mg/mL. Thus, the bactericidal capacity of CuHS is maintained even under extreme temperature exposure. This result is especially important because, in principle, it makes the future production of composites using the extrusion method possible, which has more analogy to the processes used in the packaging industry.

### 3.2. Characterization of the Composite Films

**Figure 3:**
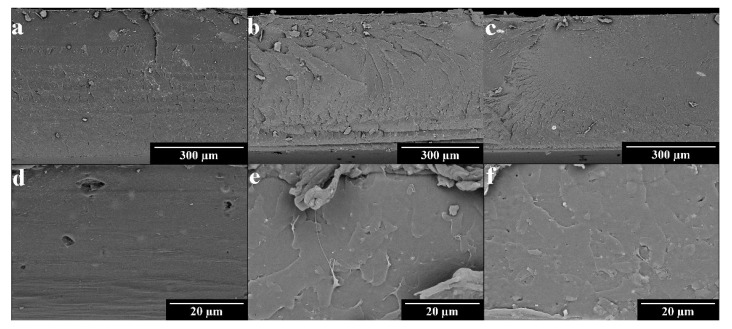
FSEM images of LDPE composites. (**a**) 0.1% CuHS; (**b**) 0.3% CuHS; (**c**) 0.5% CuHS and PLA composites; (**d**) 0.1% CuHS; (**e**) 0.3% CuHS; and (**f**) 0.5% CuHS.

The distribution of the particles in the polymeric matrices was studied with FESEM. In Figure 3, we can observe the presence of the CuHS particles in the cross-sectional cuts made in the films. A more homogeneous distribution of the CuHS is observed in the films with a PLA polymeric matrix (Figure 3d–f), which is due to the process used to obtain these composites that involved the use of an ultrasonic process to perform the solution casting method. This may cause a decrease in the size of the CuHS particles and the physical disintegration of the agglomerates that were initially obtained. In the case of the LDPE composites (Figure 3a–c), a higher number of aggregates of ~10–30 µm are observed. In this case, the method only involved a hot-mixing process using a double screw mixer, with no previous step in which the dispersion of CuHS in the polymer matrix is attempted to be improved. Furthermore, it is observed that as the CuHS loading is increased, the number and size of the aggregates increases. This effect will have important implications on the antibacterial properties of the films loaded with 0.5% CuHS in the LDPE matrix.

The thermal characterization of the neat matrices as well as that of the composites was carried out using TGA and DSC. Figure S4 shows the results obtained, which are also summarized in Table 1, where the decomposition temperature (Td) was obtained from the TGA, while the glass transition temperature (Tg), cold crystallization temperature (Tc), and melting temperature (Tm) were determined with the DSC. The presence of CuHS does not seem to significantly affect the thermal properties of the polymeric matrices due to the similarity of the parameters analyzed for neat LDPE and PLA, and to those found in the literature [13,38].

However, in the PLA samples, a slight decrease in Td is observed in the TGA. In addition, the samples with 0.3 and 0.5% CuHS show a second decomposition process at 291 and 282 °C, respectively. It should be noted that the additional loss observed in these samples seems to be accentuated as the salt content in the composite increases, which we consider may be due to the degradation of the PLA polymeric matrix catalyzed by the presence of the CuHS particles. This phenomenon occurs in the PLA matrix and not in the LDPE matrix due to the fact that PLA has easily hydrolysable ester groups, whose thermal degradation can be catalyzed by CuHS.

The decrease in the Td of PLA in the presence of nanoparticles has already been reported for other systems [6,39]. This effect is attributed to the local heating that can be induced by the nanoparticles due to their small size and subsequent high specific surface area.

The mechanical properties of the films were determined from the analysis of the stress vs. strain curves. The results obtained are shown in Table 2. The CuHS added to the LDPE matrix does not seem to affect the maximum stress and strain values or the modulus of elasticity with respect to the neat LDPE. In addition, the modulus values obtained are in correspondence with other works [40,41,42]. The similarity between the curves for the pure LDPE and its composites is shown in Figure S5a.

For the PLA samples, there is a tendency for the maximum stress to increase (>10 MPa) and the maximum strain to decrease, and in turn, there is an increase in the modulus of elasticity, observed by the statistical difference of the results (Table 2 and Figure S5b). This type of behavior is similar to the trend observed in PLA composite films with MXene, reported previously [6]. This trend is related to an increase in the brittleness of the composites due to low interaction between the PLA and the nanofiller, causing the CuHS particles to act as voids in the polymer matrix and producing a concentration of stresses [6,43,44].

### 3.3. Bactericidal Performance of the Composite Films

**Figure 4:**
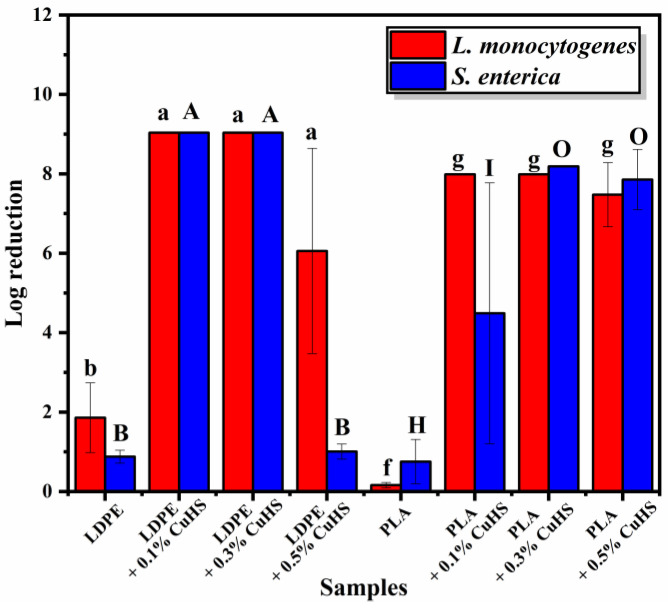
Effect of CuHS concentration on the bactericidal activity of the composite films against *S. enterica* and *L. monocytogenes*. The error bars show the standard deviation for the three to five independent samples of the composites, and three replicates for the neat polymers. Similar letters/symbols mean no statistical significance, while different letters/symbols mean statistical significance. The initial concentration of both bacteria was 10^9^ CFU/mL.

The bactericidal capacity of the films is shown in Figure 4. When evaluating the films with an LDPE matrix, it can be seen that there is a 100% reduction in the composites with 0.1 and 0.3% against both bacteria, corresponding to a reduction of ~9 log units for *S. enterica* and ~8.8 for *L. monocytogenes,* respectively. A further increase of CuHS to 0.5 wt.% displayed a different behavior, in which the activity was less effective with a reduction of only 1 log unit for *S. enterica* (no significant difference in comparison to neat LDPE) and 5.8 for *L. monocytogenes*.

We speculate that this drop is probably due to a heterogeneous dispersion of the CuHS filler in the LDPE via the melt-extrusion process, which resulted in the formation of CuHS aggregates. This would cause a decrease in the surface area/volume ratio of the particles; thus, limiting their bactericidal properties.

In the case of the composite films with a PLA matrix, whose particle dispersion is much better due to the manufacturing method, an almost total reduction is observed with the three concentrations of CuHS tested (0.1, 0.3 and 0.5%) against *L. monocytogenes*. For *S. enterica*, a concentration of 0.3% CuHS in the PLA matrix was needed to achieve a 100% reduction (~8 log units), since in the case of the composite with 0.1% CuHS, only a reduction of 5 log units was achieved.

A comparison with the antibacterial activity using the surface contact test method of composite films with other bactericidal inorganic fillers such as silver, titanium dioxide, or copper (Table 3), shows that the log reduction obtained in our study was increasingly better.

It is of great interest to study whether the bactericidal capacity of the composite films has longevity, so that the potential packaging made from them can also be reused. For this test, the loading content of 0.3 wt.% for the PLA/CuHS and 0.1 wt.% for the LDPE/CuHS were selected since it corresponded to the lowest content that demonstrated the highest bactericidal activity.

As observed in Figure S6, in the LDPE composite films, the first use had a 100% bactericidal activity against both bacteria. The activity dropped after the second and third use of the *S. enterica* bacteria. For *L. monocytogenes*, a different behavior is observed, where almost 100% activity is still maintained in the second use, suffering a further loss in the third use. In the PLA composite films, for *S. enterica,* the first use provided 100% activity, whereas the activity was reduced significantly in the second use. A 100% bactericidal behavior was observed after the first and second use for *L. monocytogenes*.

Overall, *L. monocytogenes* is more susceptible to the produced composite films, whereas *S. enterica* demonstrated higher resistance. According to the results, for both LDPE/CuHS and PLA/CuHS, the bactericidal activity tends to be lost after the first use in the case of *S. enterica* but it is maintained after at least two uses for *L. monocytogenes*.

The mechanisms by which Cu_2_(OH)_3_NO_3_ exerts its bactericidal action are still under study [13,33]. Videira-Quintela et al. comment that the bactericidal activity of CuHS is mainly due to the release of Cu(II) ions [13,33]. In this sense, we further corroborated that the activity was mainly due to the Cu(II) ions by determining the MBC for copper(II) nitrate and sodium nitrate against *L. monocytogenes* and *S. enterica*.

In this way, clarifying the bactericidal action of CuHS against the bacteria studied in this work was attempted, which is fundamentally due to the migration of Cu(II) ions and not the presence of nitrate ions, also known to possess bactericidal proprieties [51]. It was observed that NaNO_3_ does not present bactericidal activity against the studied bacteria. However, copper(II) nitrate showed an MBC of 2 and 1.5 mg/mL against *L. monocytogenes* and *S. enterica,* respectively, which were quite similar to the values obtained when determining the MBC of CuHS.

The mechanism of antimicrobial activity of copper(II) ions is closely related to their ability to interact with negatively-charged surfaces such as the membrane and cell wall of bacterial cells. This interaction affects the different biochemical processes in bacteria, causing cell death [12]. This could explain the differences in the bactericidal power of copper against different pathogens because it will depend on the characteristics of the cell membrane and the wall of the microorganism in question. On the other hand, the damage to the plasma membrane caused by the presence of Cu^2+^ can also be due to the oxidative stress produced by the release of reactive oxygen species (ROS). Scattareggia et al. shows the Cu^2+^/Cu^+^ redox-active mechanism for the generation of cytotoxic ROS [5]. 

### 3.4. Migration

Due to the potential bactericidal properties of the films with 0.3% CuHS loading, both with an LDPE and PLA matrix, and the importance of Cu(II) ion migration in the antibacterial mechanism, copper migration assays of these composites were performed. Moreover, this assay is especially important because the release of copper is regulated in applications related to food packaging [5,13,33].

**Figure 5:**
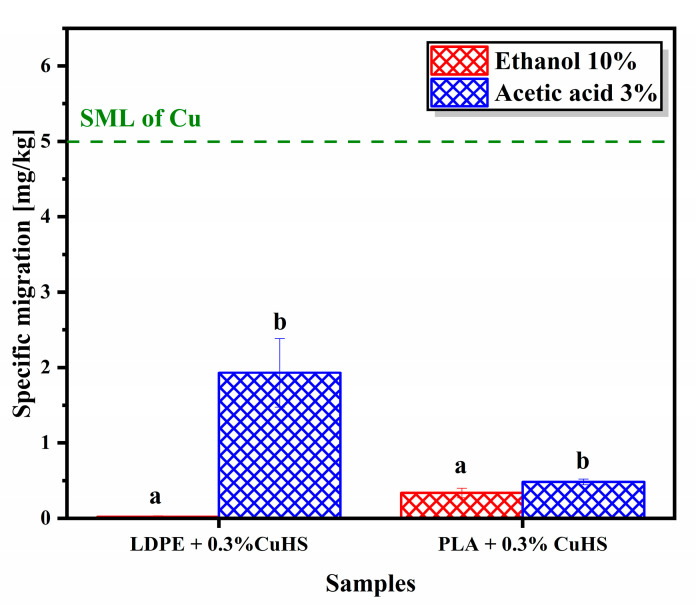
Specific migration of copper from the LDPE and PLA samples with 0.3% CuHS in the food simulants ethanol (10% *v*/*v*) and acetic acid (3% *v*/*v*). The SML takes into account the standard value of 5 mg/kg of Cu for a 6 dm^2^/kg sample testing, according to the Regulation 10/2011 [34]. The error bars show the standard deviation of the five independent samples tested. Similar letters/symbols mean no statistical significance, while different letters/symbols mean statistical significance.

Figure 5 shows the migration of Cu in the composites analyzed in the food simulants (ethanol 10% *v*/*v* and acetic acid 3% *v*/*v*). The highest copper migration, both in an LDPE matrix and PLA, occurs in an acidic medium (statistically significant). This result agrees with the literature, in which a higher dissolution/migration of metals in acidic pH is appreciated [5]. The highest value of specific migration was witnessed in LDPE films with 0.3% CuHS in an acetic acid simulant, which was 1.93 mg/kg. Still, we can observe that all values are below the specific migration limit (SML) for food contact materials, which in the migration assay is 5 mg/kg [5,13]. In addition, we consider that due to the polarity of the polymeric matrix, there is a higher interaction of CuHS particles with PLA (terminal hydroxyl groups and carbonyl groups) than with LDPE, which explains why there is a higher migration of Cu_2_(OH)_3_NO_3_ in the LDPE matrix.

### 3.5. Cytotoxicity

Although the migration of Cu is below the permitted limits, cytotoxicity tests were also performed in order to further support safety with respect to the possible use of CuHS as an additive to develop antibacterial contact surfaces for food packaging applications. The cytotoxicity assay was carried out with the HeLa cell line, which are cells derived from cervical-uterine cancer.

**Figure 6:**
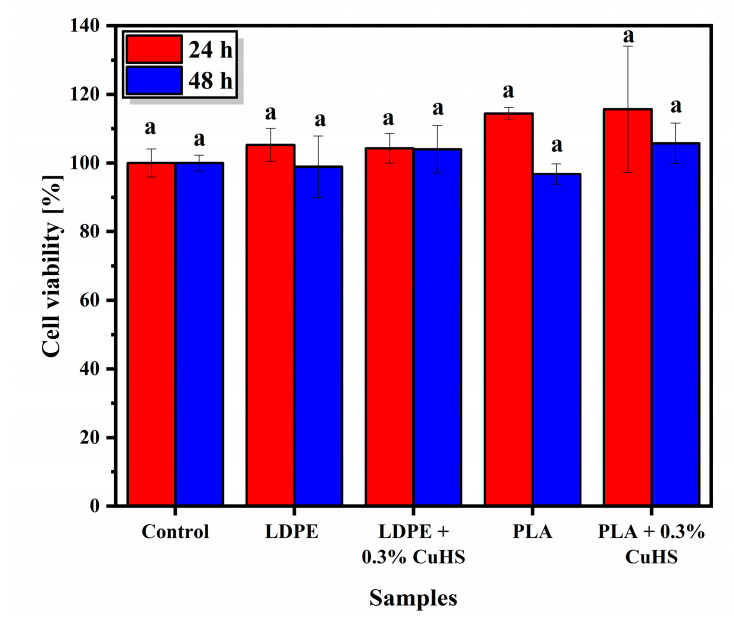
Effect of neat LDPE and PLA films and their corresponding composite film with 0.3% CuHS on HeLa-type cell viability. The error bars show the standard deviation of the four replicates performed. Similar letters/symbols mean no statistical significance, while different letters/symbols mean statistical significance.

In Figure 6, the results obtained from the cytotoxicity assay for both the LDPE and PLA matrix and their respective composites loaded with 0.3% CuHS show a cell viability similar to the control, both at 24 and 48 h after the assay (no statistical difference observed). This means that the number of live or metabolically active cells is practically the same with the presence or absence of the composites. If we consider that a cell viability above 70% means that the samples tested are not cytotoxic, then the safety of our systems against the HeLa cell line is clearly demonstrated [6,52].

## 4. Conclusions

In this work, the effectiveness of Cu_2_(OH)_3_NO_3_ as an inorganic filler in both LDPE and PLA matrices for the production of active materials with antibacterial properties has been demonstrated. The thermal properties of LDPE-based composites are practically unaffected by the addition of CuHS at the concentrations tested. In the case of PLA composites, a mass loss just before the polymer degradation temperature is observed, which could be due to the degradation of the CuHS itself. The study of the mechanical properties showed a greater influence on the addition of CuHS in PLA-based composites, where, as the loading of CuHS increases, an increase in the brittleness of the material with respect to pure PLA is observed. Composites derived from both polymeric matrices showed significant bactericidal properties against *L. monocytogenes* and *S. enterica* with 0.3% CuHS being the optimum concentration for a more efficient bactericidal action. To support its future use as an additive for food packaging applications, the Cu(II) migration and cytotoxicity of these samples were evaluated. In all cases, the specific migration of Cu(II) was below the allowed limits and the composite films were not cytotoxic against the HeLa cell line. All mentioned results corroborate the potential of copper(II) hydroxy nitrate as an excellent additive for the development of bactericidal contact surfaces for food packaging applications. The ultimate goal is to evaluate the effectiveness of the composite films on the shelf, and to extend the life of perishable foods with large environmental footprints, such as red meat, poultry, and fish.

## Figures and Tables

**Table 1:** Results obtained from thermal analysis using the TGA and DSC of neat and composite films.

Sample	T_d,onset_ [°C]	T_g_ [°C]	T_c,onset_ [°C]	T_m,onset_ [°C]
LDPE	457	-	-	103
LDPE + 0.1% CuHS	455	-	-	103
LDPE + 0.3% CuHS	460	-	-	103
LDPE + 0.5% CuHS	455	-	-	103
PLA	356	57	105	143
PLA + 0.1% CuHS	344	58	108	143
PLA + 0.3% CuHS	349	57	106	143
PLA + 0.5% CuHS	344	59	108	146

**Table 2:** Thickness and width of the specimens tested in the mechanical analysis, as well as the main results obtained from this test. The standard deviation is for five independent samples. Similar letters/symbols mean no statistical significance, while different letters/symbols mean statistical significance.

Sample	Thickness [mm]	Width [mm]	σ_max_ [MPa]	ε_max_ [%]	E [GPa]
LDPE	0.430 ± 0.007 ^c^	1.9 ± 0.1 ^a^	10.6 ± 0.3 ^a^	151 ± 7 ^a^	0.064 ± 0.009 ^a^
LDPE + 0.1%CuHS	0.438 ± 0.004 ^bc^	1.65 ± 0.08 ^a^	10.7 ± 0.5 ^a^	153 ± 48 ^a^	0.061 ± 0.008 ^a^
LDPE + 0.3%CuHS	0.481 ± 0.007 ^a^	1.8 ± 0.1 ^a^	10.4 ± 0.2 ^a^	159 ± 31 ^a^	0.065 ± 0.007 ^a^
LDPE + 0.5%CuHS	0.445 ± 0.005 ^b^	1.9 ± 0.2 ^a^	10.5 ± 0.3 ^a^	151 ± 34 ^a^	0.059 ± 0.007 ^a^
PLA	0.053 ± 0.006 ^A^	2.0 ± 0.1 ^A^	40 ± 10 ^B^	15 ± 6 ^A^	2.5 ± 0.4 ^B^
PLA + 0.1%CuHS	0.06 ± 0.02 ^A^	1.98 ± 0.05 ^A^	63 ± 12 ^A^	13 ± 6 ^AB^	3 ± 1 ^AB^
PLA + 0.3%CuHS	0.045 ± 0.005 ^A^	2.1 ±0.1 ^A^	52 ± 7 ^AB^	6 ± 3 ^B^	3.6 ± 0.2 ^A^
PLA + 0.5%CuHS	0.044 ± 0.003 ^A^	2.1 ± 0.1 ^A^	59 ± 6 ^A^	6 ± 2 ^B^	3.6 ± 0.2 ^A^

**Table 3:** Polymeric matrix/filler antibacterial systems (maximum values) reported in the literature using the surface contact test method.

Filler	Polymer	Bacteria	Log Reduction	Ref.
Montmorillonite/Resveratrol	LDPE	*S. aureus*	~5	[45]
TiO_2_NPs	Gelatin	*S. aureus* *E. coli*	~2.2 ~1.8	[46]
CuNPs AgNPs	PE	*L. monocytogenes*	~6 ~6	[47]
CuO	Poly(3-hydroxybutyrate-co-3-hydroxyvalerate)	*L. monocytogenes* *S. enterica*	~6 ~4	[48]
CuNPs	PLA	*Pseudomonas spp*	~1.4	[17]
AgNPs	Polypropylene	*S. aureus* *E. coli*	~1.6 ~6	[49]
MXene	PLA	*L. monocytogenes* *S. enterica*	~6 ~5.2	[6]
SiO_2_/Fe/Tea polyphenols	PLA	*S. aureus* *P. aeruginosa* *S. enterica*	~4 ~4 ~4	[50]
CuHS	LDPE PLA	*L. monocytogenes* *S. enterica*	~8/9	This work

## Data Availability

The data that support the findings of this study are available from the corresponding authors upon reasonable request.

## Supplementary Materials

The following supporting information can be downloaded at: https://www.mdpi.com/article/10.3390/polym15071661/s1, Figure S1. Diffractograms (XRD) of (a) CuHS, (b) CuHS subjected to 120 °C, (c) CuHS subjected to 140 °C and (d) CuHS subjected to 160 °C. The heat treatments were carried out for 1 h at the declared temperature and then XRD was performed. Figure S2. Thermal analysis of CuHS using (a) TGA and DTA, (b) DSC. Figure S3. MBC against *L. monocytogenes* of CuHS subjected to (a) 140 °C and (b) 160 °C. The numbers represent the concentrations that were analyzed: 1 corresponds to 0.5 mg/mL, 2 to 1 mg/mL, 3 to 2 mg/mL, and 4 to 3 mg/mL. Figure S4. Thermal properties of films: (a) TGA of LDPE and its composites, (b) TGA of PLA and its composites, (c) DTA of LDPE and its composites, (d) DTA of PLA and its composites, (e) DSC of LDPE and its composites, and (f) DSC of PLA and its composites. Figure S5. Stress vs. strain curves for films of (a) LDPE and its composites, (b) PLA and its composites. Figure S6. Repeatability test to determine the antibacterial properties of (a) LDPE matrix, (b) PLA matrix films. LDPE-I and LDPE + 0.1% CuHS-I depicts antibacterial properties in the first use; LDPE-II and LDPE + 0.1% CuHS-II in the second use; and LDPE-III and LDPE + 0.1% CuHS-III in the third use. For PLA matrix samples, PLA-I and PLA +0.3%-I describe the antibacterial properties of these films in the first use, and PLA-II and PLA +0.3%-II corresponds to the second use. Table S1. Results obtained using the EDX analysis of the synthesized CuHS.
