# Supplementary material for: Capability of Copper Hydroxy Nitrate (Cu2(OH)3NO3) as an Additive to Develop Antibacterial Polymer Contact Surfaces: Potential for Food Packaging Applications
Source: Polymers (Basel). 2023 Mar 27;15(7):1661. doi: 10.3390/polym15071661 (PMC10096628; doi:10.3390/polym15071661)
Supplement: Supplementary file 1 [file polymers-15-01661-s001.zip › polymers-2254742-supplementary.pdf]

polymers

Supplementary Information

Capability of Copper Hydroxy Nitrate ($Cu_2(OH)_3NO_3$) as an Additive to Develop Antibacterial Contact Surfaces: Potential for Food Packaging Applications

Xiomara Santos [1], Juana Rodríguez [2], Francisco Guillén [2], Javier Pozuelo [1], J.M. Molina-Guijarro [2], Diogo Videira-Quintela [3,*], and Olga Martín [1,*]

[1] Escuela Politécnica Superior, Departamento de Ciencia e Ingeniería de Materiales e Ingeniería Química, Universidad Carlos III de Madrid, Avenida Universidad 30, 28911 Leganés, Spain
[2] Facultad de Farmacia, Departamento de Biomedicina y Biotecnología, Universidad de Alcalá, Ctra. Madrid-Barcelona km 33.6, 28871 Alcalá de Henares, Spain
[3] Facultad de Farmacia, Departamento de Química Analítica, Química Física e Ingeniería Química, Universidad de Alcalá, Ctra. Madrid-Barcelona km 33.6, 28871 Alcalá de Henares, Spain
* Correspondence: diogo.videira@uah.es (D.V.-Q.); martinc@ing.uc3m.es (O.M.)

Table S1. Results obtained by EDX analysis of the synthesized CuHS.

Element	Weight %	Atomic %	Error %
N K	8,85	16,01	11,26
O K	40,21	63,68	8,17
Cu K	50,94	20,31	3,27

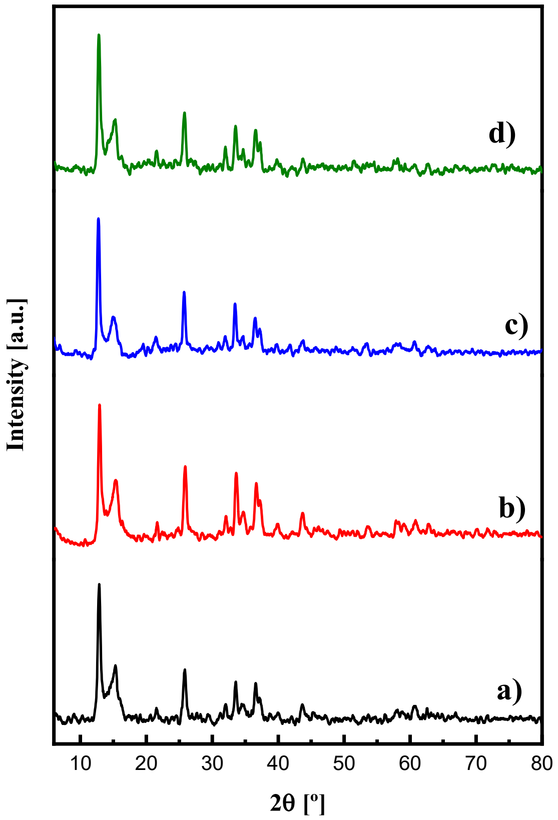

Figure S1. Diffractograms (XRD) of a) CuHS, b) CuHS subjected to 120 °C, c) CuHS subjected to 140 °C and d) CuHS subjected to 160 °C. The heat treatments were carried out for 1 hour, at the declared temperature and then XRD was performed.

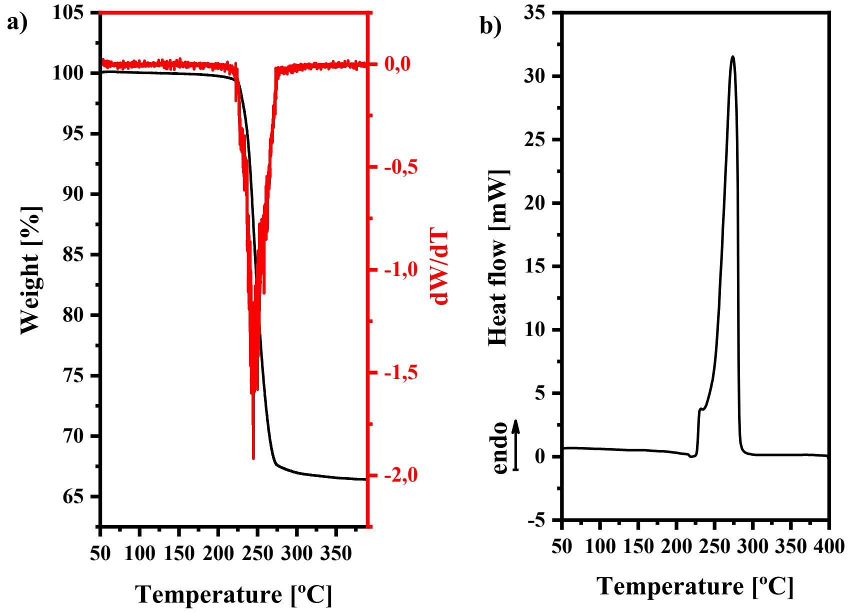

Figure S2. Thermal analysis of CuHS by a) TGA and DTA, b) DSC.

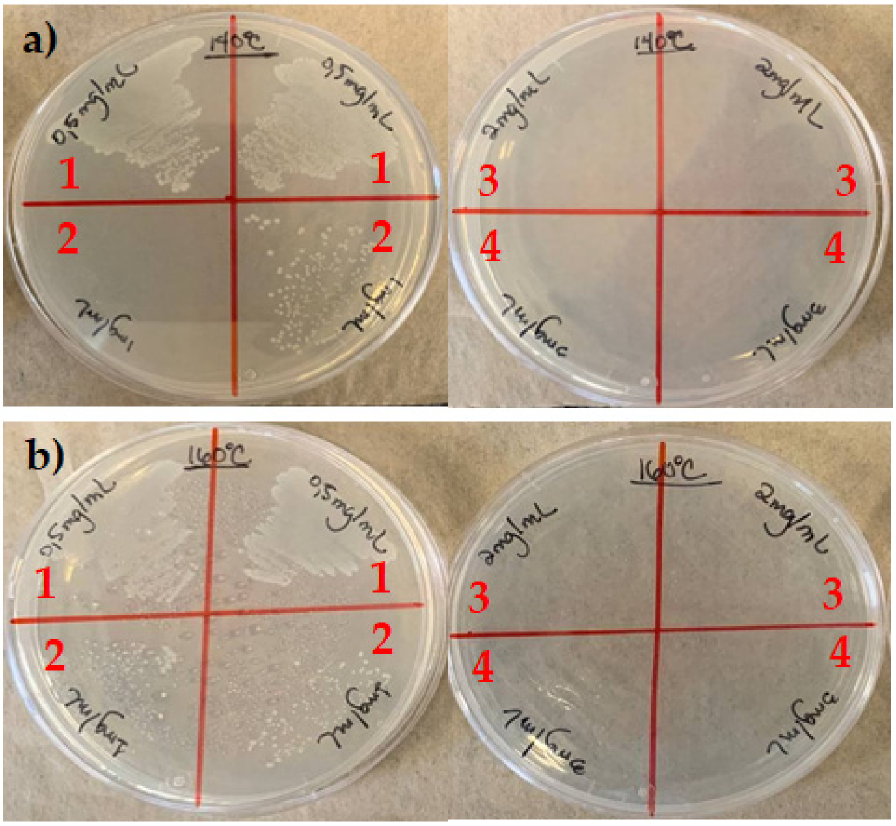

Figure S3. MBC against *L. monocytogenes* of CuHS subjected to a) 140 ºC and b) 160 ºC. The numbers represent the concentrations that were analyzed: 1 corresponds to 0.5 mg/mL, 2 to 1 mg/mL, 3 to 2 mg/mL and 4 to 3 mg/mL.

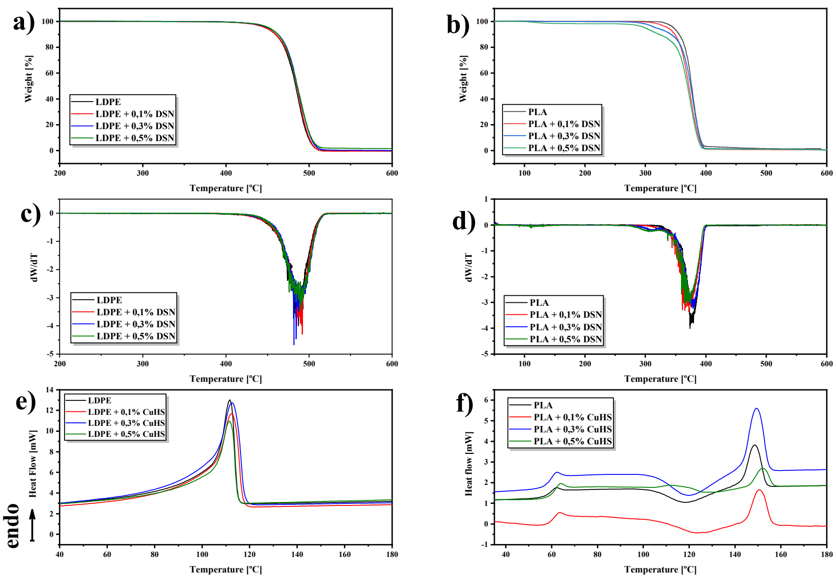

Figure S4. Thermal properties of films. a) TGA of LDPE and its composites, b) TGA of PLA and its composites, c) DTA of LDPE and its composites, d) DTA of PLA and its composites, e) DSC of LDPE and its composites and f) DSC of PLA and its composites.

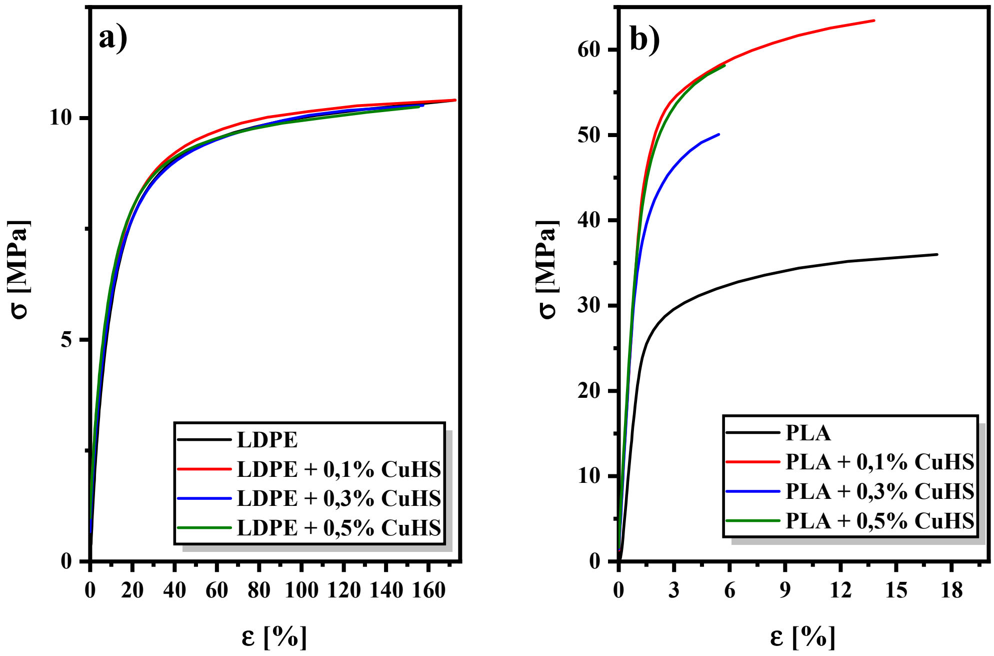

Figure S5. Stress vs. strain curves for films of a) LDPE and its composites, b) PLA and its composites.

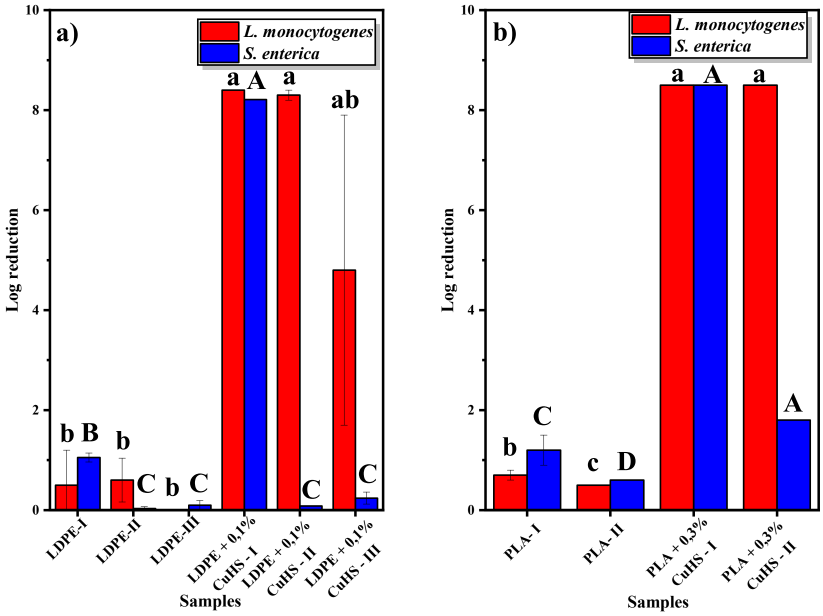

Figure S6. Repeatability test to determine antibacterial properties of a) LDPE matrix, b) PLA matrix films. LDPE-I and LDPE + 0.1% CuHS-I depicts antibacterial properties in the first use, LDPE-II and LDPE + 0.1% CuHS-II in the second use and LDPE-III and LDPE + 0.1% CuHS-III in the third use. For PLA matrix samples, PLA-I and PLA +0.3%-I describe the antibacterial properties of these films in the first use and PLA-II and PLA +0.3%-II corresponds to the second use.
